# Micro-fragmented adipose tissue cellular composition varies by processing device and analytical method

**DOI:** 10.1038/s41598-022-20581-1

**Published:** 2022-09-27

**Authors:** Valerie Greenwood, Peter Clausen, Andrea M. Matuska

**Affiliations:** Arthrex Inc, One Arthrex Way, Naples, FL 34108 USA

**Keywords:** Stem-cell research, Adult stem cells, Mesenchymal stem cells

## Abstract

Autologous adipose-derived biologics are of clinical interest based on accessibility of adipose tissue, a rich source of progenitor and immunomodulatory cells. Micro-fragmented adipose tissue (MFAT) preserves the cellular niche within intact extracellular matrix, potentially offering benefit over enzymatically-liberated stromal vascular fraction (SVF), however lack of standardized analyses complicate direct comparison of these products. In this study, MFAT from LipoGems® and AutoPose™ Restore systems, which utilize different washing and resizing methods, was analyzed for cellular content using different techniques. Flow cytometry was performed on SVF, with or without culture, and on the adherent cell population that naturally migrated from undigested MFAT. Cytokine release during culture was also assessed. SVF contained more diverse progenitor populations, while MFAT outgrowth contained lower cell concentrations of predominantly mesenchymal stromal cells (MSC). MSCs were significantly higher in MFAT from the AutoPose System for all analyses, with increased cytokine secretion characterized by high levels of anti-inflammatory and low to non-detectable inflammatory cytokines. These results demonstrate that cellularity depends on MFAT processing methods, and different techniques can be employed to evaluate graft cellularity. Comparisons of cell concentrations determined via these methods could be used to better interpret inter-study variability.

## Introduction

Adipose tissue is a precursor to a wide spectrum of regenerative therapies due to its accessibility and abundant cellular content comprised of stromal progenitors such as adipose-derived stromal cells (ASCs, similar to mesenchymal stromal cells, MSCs), pericytes, endothelial, and immunoregulatory cells including white blood cells (WBCs)^1^. The extracellular matrix (ECM) is comprised of collagens, laminin, fibronectin, and glycosaminoglycans which offer inherent supportive properties as a scaffold^2^.

The term stromal vascular fraction (SVF) refers to the pelleted cell component of adipose tissue independent of the ECM and adipocytes. This can only truly be isolated via enzymatic means, however some research has investigated mechanical methods of SVF isolation which are inefficient in comparison^3^. Furthermore, enzymatic digestion methods are time consuming, expensive, and typically excluded from regulatory-approved minimal manipulation guidelines. Alternatively, adipose can be resized into micro-fragmented adipose tissue (micro-fat or MFAT) that retains structural collagens and a microenvironmental-niche which may offer benefits over SVF alone including cushioning, support, and enhanced graft fixation/retention after treatment^4^. Graft size can be labeled as a nano-, micro-, or milli-fragmented adipose tissue depending on harvesting and resizing methods^5^.

Differences in harvesting techniques and location, processing methods such as enzymatic digestion, mechanical emulsification, washing, or centrifugation, and donor variation may lead to variable MFAT product output^5,6^. Lack of standardized characterization methods and interchangeable terminology also complicate comparing results among different laboratory studies^7^.

The purpose of this study was to develop and compare analytical methods to characterize MFAT produced using two commercially available FDA-cleared devices, the AutoPose™ Restore and Lipogems® Systems. While both systems are designed to produce MFAT from lipoaspirate, without centrifugation, processing methods differ as AutoPose™ Restore is a double-syringe system and Lipogems® is a closed-loop system. The tissue processed using Lipogems is resized initially through a 2 mm filter, followed by a second resizing through a 1 mm filter as the final processing step. This contrasts with the AutoPose System that relies on a single step resizing through a 0.8 mm filter. Sample washing steps are also different between the two systems in that Lipogems utilizes a continuous flow of a large volume (> 500 cc) saline wash whereas washing in the AutoPose System is accomplished with sequential introduction and decanting of 2 × 15 cc volumes of saline. The hypothesis was that differences in washing and resizing protocols between the systems would lead to different product compositions.

## Materials and methods

### Ethics approval and consent to participate

Lipoaspirate samples were obtained from patients undergoing elective liposuction with informed written consent. Ethical approval of the study protocol was received from an AAHRPP-accredited Institutional Review Board (Salus, Austin, TX, IRB #1072). All methods were performed in accordance with the relevant guidelines and regulations of the ethics committee.

### Lipoaspirate collection

Lipoaspirate was harvested from five donors (n = 5) using vacuum aspiration with a cannula ranging from 3 to 5 mm. All donors were female with an average age of 53 ± 16, ranging from 28 to 70 years of age, and an average body mass index (BMI) of 27 ± 5, ranging from 24 to 34. Lipoaspirate was obtained from various locations of the body including the inner and outer thighs, mid and lower back, flanks, and abdomen. After collection, lipoaspirate samples were stored at room temperature and processed within 8 h of harvest. Samples were thoroughly mixed throughout allocation to the different systems and during processing to ensure homogeneity. Weights, in addition to volume, were monitored throughout processing to account for any error in volumetric distribution of the lipoaspirate.

### AutoPose procedure

Harvested lipoaspirate was injected through the AutoPose™ Refresh harvesting cannula (1.5 mm × 1.0 mm hole size) to mimic harvesting instructions for use prior to processing with the AutoPose™ Refresh syringe (Arthrex, Naples FL). The weight of the AutoPose syringe was recorded and 30 cubic centimeter (cc) of resized decanted lipoaspirate was injected into the AutoPose double-syringe system through the provided luer transfer and reweighed. The decanted adipose was washed twice by injecting 15 ml of sterile saline (0.9% Sodium Chloride, VWR, Radnor, PA) into the AutoPose syringe, inverting for 10 s, allowing to gravimetrically separate for 3 min, and decanting completely. After washing, the outer flanges were pushed to micro-fragment the adipose tissue through an 800 µm filter into the AutoPose inner syringe. The weight of the inner syringe containing the micro-fragmented adipose product was recorded before and after collection.

### Lipogems procedure

Harvested lipoaspirate was injected through a Lipogems® harvesting cannula (5.0 mm × 1.7 mm hole size) to mimic harvesting instructions for use with the Lipogems device (LIPOGEMS International, Norcross, GA). The weight of an empty Lipogems syringe was recorded and 30 cc resized decanted adipose was added to be processed with the system. The system was connected to a bag containing sterile saline (0.9% Sodium Chloride, VWR, Radnor, PA), and unclamped to allow the canister to pre-fill completely with saline and then rotated. The syringe containing the decanted adipose was attached and 15 cc was injected into the canister of the closed-loop Lipogems® system. The system was unclamped to allow flow of saline to rinse adipose until the saline waste appeared clear. With both tubes clamped, the canister was shaken vigorously for 30 s intervals and clamps were re-opened for 10 s or until the saline rinsed clear. The shaking was repeated for a total of three times. The canister was rotated again to collect product. Saline was drawn into a lower syringe connected to the canister and then pushed into the canister to force the adipose tissue through another resizing filter into the upper product syringe. This entire process was repeated using the remaining 15 cc of decanted adipose. The final combined micro-fragmented adipose product was decanted after 3 min to allow separation of saline from the product and a final weight was recorded.

### Cell characterization

**Figure 1:**
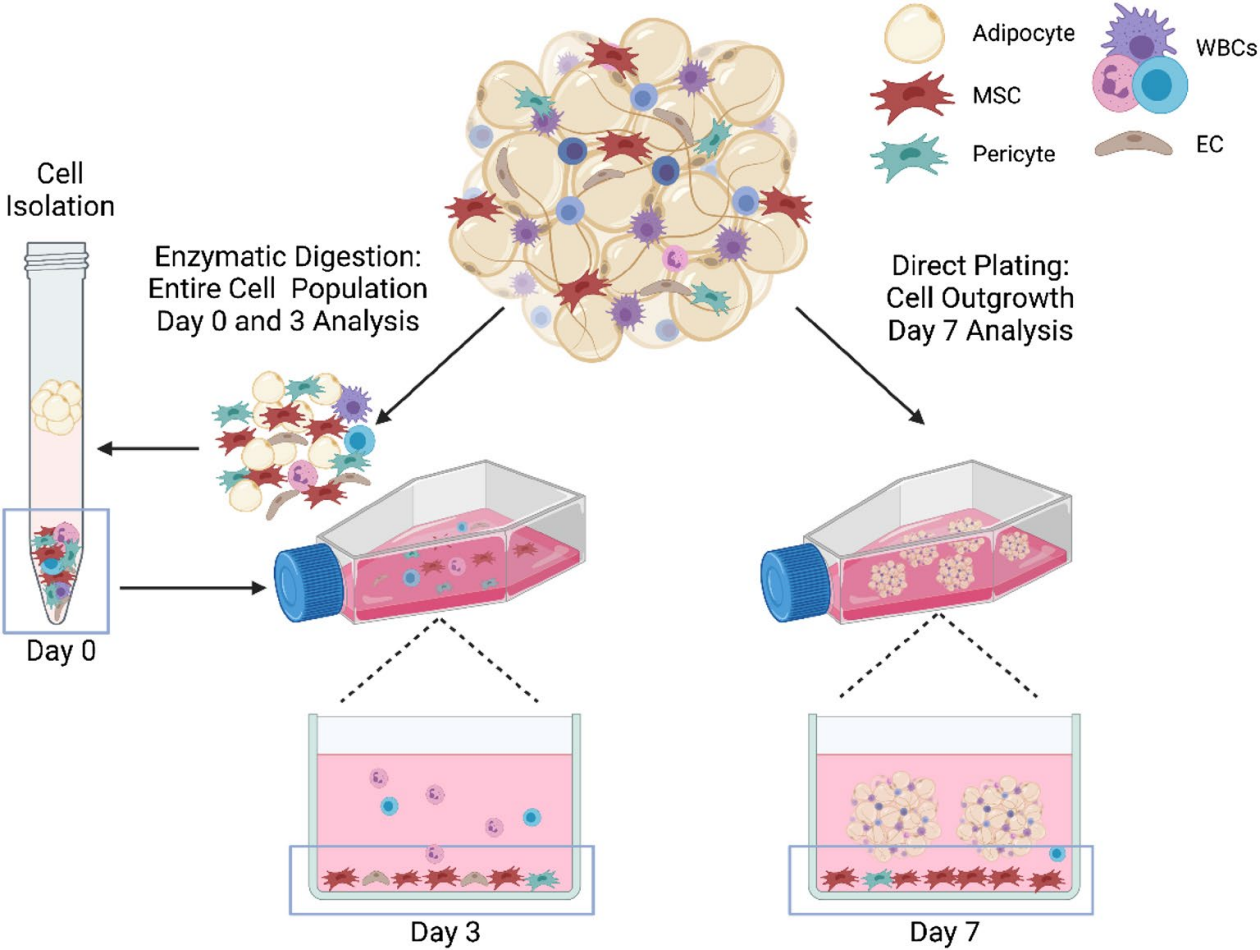
Schematic demonstrating cell population isolated and characterized from MFATs. Enzymatic dissociation was used to isolate a cell pellet for initial (Day 0) and early culture (Day 3) analysis. Direct plating of MFAT was used to evaluate cell outgrowth from the tissue (Day 7). Figure created with www.BioRender.com.

Cellular composition of MFAT products was evaluated through several different methods using a combination of flow cytometry and cell culture at different timepoints (Fig. 1). Prior to analysis, triplicate, 0.5 cc sample aliquots of each MFAT preparation were weighed to obtain exact mass values to be used in normalization of data on a per gram basis for each of the following assays described. The re-sized adipose samples were cultured as undigested MFAT and in parallel cultured after enzymatic digestion to liberate cells from extracellular matrix.

To enzymatically digest the MFAT, 0.5 cc samples in triplicate were incubated in 15 ml conical tubes with 5 ml of 0.25 mg/ml of Liberase TL (Sigma Aldrich, St. Louis, MO) in Dulbecco’s Phosphate-Buffered Saline (DPBS) for 30 min at 37 °C on a tube rocker. Samples were centrifuged at 900×*g* for 5 min and supernatant was decanted. The cell pellet was resuspended in 5 ml of complete Dulbecco’s Modified Eagle Medium/Nutrient Mixture F-12 (DMEM/F-12), containing 10% Fetal Bovine Serum and 1% Penicillin–Streptomycin (Gibco, Waltham, MA), and 200 µl was set aside for Time 0 analysis. The remaining cell suspension was cultured in T-25 flasks for 3 days at 37 °C in a humidified CO_2_ incubator before subsequent analysis. After 24 h, flasks were rinsed with DPBS and media was changed to remove nonadherent cells.

For direct culture of MFAT, 0.5 cc of adipose product was pipetted in triplicate into T-25 flasks, 5 ml of complete DMEM/F-12, as described above, was added, and samples were gently dispersed for even distribution on the flask surface. Flasks were incubated at 37 °C for 7 days, with no rinsing or media change to disturb cell outgrowth from the tissue. After the culture duration, conditioned media for cytokine analysis was collected from each flask into 15 ml conical tubes and centrifuged at 600×*g* for 5 min. Supernatant devoid of MFAT or cells was collected and stored frozen at − 80 °C in cryovials until enzyme-linked immunosorbent assay (ELISA) analysis could be performed.

### Flow cytometry

Flow Cytometry was performed on a Beckman Coulter CytoFLEX (Brea, CA) with 3 lasers (Violet-Blue-Red) and 9 activated bandpass channels. Data analysis was performed in the CytExpert software (Beckman Coulter, Brea, CA). An 8-color flow panel was developed to evaluate cell populations in the MFAT samples (Table 1). All antibodies and reagents including cell staining buffer and red blood cell (RBC) lysis solution were acquired from BioLegend (San Diego, CA) except for UltraComp eBeads (Invitrogen, Waltham, MA) which were used to perform compensation.

**Table 1:** Summary of markers used and associated cell types.

Marker	Flourochrome	Clone	Ratio	Associated cell types
DRAQ5	DRAQ5	N/A	1:200	Nucleated cell
DAPI	DAPI	N/A	1:100	Viability exclusion
CD45	PerCP/Cy5.5	2D1	1:20	WBC/HPC
CD34	BV785	561	1:100	Adipose derived-MSC (−), HPC, EC
CD146	PE/Cy7	SHM-57	1:100	Vessel associated (Pericyte, EC) (+/−)
CD31	APC/Cy7	WM59	1:20	EC
CD90	PE	5E10	1:100	MSC
CD73	APC	AD2	1:40	MSC
CD105	FITC	43A3	1:40	MSC (+)

All antibodies were purchased from BioLegend (San Diego, CA) and respective catalog numbers are shown.

*MSC* mesenchymal stromal cell, *HPC* hematopoietic progenitor cell, *EC* endothelial cell.

Symbols indicate expression (−) lost or (+) gained in culture^1,6,8–11^.

To remove culture adherent cells for flow analysis, TrypLE (Gibco, Waltham, MA) was used to detach adherent cells and cell suspensions were resuspended in a known amount of flow cytometry cell staining buffer to be able to perform total cell count calculations. Total nucleated cell (TNC) count was assessed at each time point using DRAQ5 (1:200 dilution, 20-min incubation, 4 °C). These analyses were performed independent of the multicolor characterization panel, to avoid interference with far red flourochromes and to minimize any manipulation of cell suspensions that could affect the accuracy of the TNC count. These suspensions were analyzed on the flow cytometer at a collection rate of 60 µl/min with a 60 s collection time. TNC/g was calculated from DRAQ5+ TNC event counts normalized to the weight of the samples.

In an independent sample tube, CD marker full panel analysis was performed to determine the proportion of each cell population. Briefly, cell suspensions were incubated with RBC lysis buffer to lyse RBCs, resuspended in 100 µl cell staining buffer, and incubated with antibodies for 20 min at 4 °C. Samples then were washed and stained with DAPI (1:100) for 5 min before data acquisition. Additional cell suspensions from parallel cultures were pooled to produce a sample for fluorescence minus one (FMO) controls to determine gating strategies for the multicolor stained samples.

For analysis of cultured samples, forward and side scatter areas were used to isolate cells, followed by exclusion of doublets and DAPI+ non-viable cells. To gate populations of interest within the viable cell population, CD45− and CD45+ cells were separated and CD45− cells were further analyzed for remaining markers dependent on the timepoint being analyzed. For example, CD34− were gated for CD146+ to determine a pericyte, while CD34+, CD146+ were termed adventitial endothelial cell (EC)/pericyte progenitors. Later timepoints used CD31 to distinguish EC (CD31+) from pericytes (CD31−). Stromal cells were further gated using CD31−, CD90+, CD73+, and CD105+ (later cultured time-points). See Supplementary Files for full gating strategies.

### Growth factor/secretome analysis

A combination of Multiplex, Quantikine, and DuoSet ELISAs (all R&D Systems, Minneapolis, MN) were performed on samples collected from Day 7 conditioned cell culture media to measure pro- and anti-inflammatory cytokines and growth factors. A cytokine panel was assayed using the MAGPIX system (Luminex, Austin, TX) and single ELISAs were assayed using an Epoch Microplate Spectrophotometer with Gen5 Analysis Software (BioTek, Winooski, VT). The following 23 cytokines and growth factors were assessed: IL-1ra, VEGF, TGF-β1, M-CSF, HGF, bFGF, PDGF-BB, IL-13, IL-33, IL-10, IL-4, TGF-α, IL-6, IL-2, IL-15, IFN-α, IL-7, IL-3, GM-CSF, TNF-α, IL-1β, IL-1α, and IFN-γ. Concentrations were normalized to each gram of MFAT cultured.

### Statistical analysis

All statistical analyses were performed using SigmaPlot 14.0 (Systat Software Inc, San Jose, CA). The aggregate mean and standard deviation (SD) were calculated for each data set (n = 5) and represented as “mean ± SD”. Based on the hypothesis that differences in processing methods between systems (i.e. the heavier washing protocol of Lipogems) would result in different MFAT compositions, it was predicted that Lipogems MFAT product would yield significantly lower cellular content compared to AutoPose. Donor samples were paired, therefore a donor paired one-tailed t-test was performed between the MFAT for Time 0, Day 3, and Day 7 cell counts and cytokine concentrations (α = 0.05, 95% CI) when assumptions of normality and equal variance were met. Therefore, the null hypothesis (H_0_) was that the cell and cytokine outputs of the devices were the same. When H_0_ was rejected (p ≤ 0.05), statistical significance was demonstrated. A nonparametric Wilcoxon Signed Rank test was performed for the only data set (Day 3 pericyte count, p = 0.625) that was shown to be not normally distributed via Shapiro–Wilk’s test. This data was represented as median (interpercentile range).

## Results

The amount of decanted fat initially added to the systems were 28.1 ± 0.7 and 28.3 ± 0.7 g in AutoPose and Lipogems, respectively. The final isolated MFAT was 20.9 ± 3.4 g and 12.0 ± 2.0 g, corresponding to a recovery of 74% and 42%, respectively. In accordance with each products instructions for use, washing the tissue in the AutoPose System was accomplished with 30 cc saline compared to 550–900 cc (731 ± 168) saline used to wash tissue in the Lipogems System.

### Cell characterization

Initial viable nucleated total cell counts obtained by enzymatic digestion of MFAT were 285 ± 122 10^3^ TNC/g of AutoPose product and 236 ± 114 10^3^ TNC/g of Lipogems product (p = 0.136, Table 2). The concentration of CD45+ WBCs, CD45−CD34− CD146+ pericytes and CD45−CD34+CD146+ adventitial EC/progenitor cells were not significantly different in samples prepared with either system. The viable TNC population was comprised of 44% ± 9% and 50% ± 9% WBCs and 9.9% ± 3.1% and 10.8% ± 2.6% adventitial ECs for AutoPose and Lipogems Systems, respectively. However, the stromal MSC population designated by CD45−CD34+CD90+CD73+ represented 27% ± 8% of the TNC population in samples prepared with the AutoPose System, as compared to 17% ± 9% obtained with the Lipogems System, and the total concentration was significantly higher (p = 0.020).

**Table 2:** Initial number of cells normalized per gram of enzymatically digested product (10^3^/g).

Cell type	AutoPose (10^3^/g)	Lipogems (10^3^/g)	p-value
Viable TNC	285 ± 122	236 ± 114	0.136
WBC: CD45+	131 ± 71	125 ± 80	0.402
Pericyte: CD45−34−146+	57 ± 34	46 ± 16	0.190
Adventitial EC/pericyte progenitor: CD45−34+146+	28 ± 10	24 ± 7	0.218
Stromal MSC: CD45−34+90+73+	74 ± 44	39 ± 25	0.020

Aggregate mean and standard deviation (SD) of data represented as “mean ± SD”.

Once this cell population was allowed 3 days in culture, the adherent and proliferative stromal MSC population (CD45−CD31−CD34+CD90+CD73+CD105+) represented 73% ± 12% and 36% ± 16% of the nucleated cell population of AutoPose and Lipogems, respectively (Table 3), with significantly more TNCs and MSCs from AutoPose sample (p = 0.040 and p = 0.012). ECs characterized by CD45−CD34+CD146+CD31+ represented a larger portion of the cell population at the 3-day time point for samples prepared with Lipogems, at 25%, compared to 11% of the population for AutoPose (p = 0.260). Small amounts of CD45+ contaminating WBCs (< 2%) were present in similar amounts in the culture (p = 0.249).

**Table 3:** Number of cells normalized per gram of enzymatically digested product 3 days after culture (10^3^/g).

Cell type	AutoPose (10^3^/g)	Lipogems (10^3^/g)	p-value
Viable TNC	351 ± 140	200 ± 74	0.040
WBC: CD45+	5.6 ± 3.9	4.2 ± 2.7	0.249
Pericyte: CD45−34–146+31−*	20 (16–46)	26 (22–34)	0.625
Endothelial cell: CD45−34+146+31+	39 ± 22	50 ± 25	0.260
Stromal MSC: CD45−31–34+90+73+105+	261 ± 118	79 ± 54	0.012

Aggregate mean and standard deviation (SD) of data represented as “mean ± SD”.

*Data set not normally distributed and represented as “median (interpercentile range)”.

The amount of direct cell outgrowth from MFAT over 7 days was an order of magnitude lower than the cells characterized post enzymatic liberation initially and at Day 3 (Table 4). Cell concentrations were 58 ± 35 × 10^3^ TNC/g of AutoPose product and 12 ± 7 × 10^3^ TNC/g of Lipogems product (p = 0.016). Outgrowth from AutoPose product resulted in a cell population overwhelmingly characterized as stromal MSCs that were CD45−CD31−CD90+CD73+CD105+, comprising 85% of the population with 15% WBC contamination, while Lipogems product resulted in a cell population consisting of mostly WBCs, at 68%, with 32% stromal MSCs. Overall WBC concentration in cultures was similar between groups (p = 0.276).

**Table 4:** Number of outgrowth cells normalized per gram of product 7 days after culture (10^3^/g).

Cell Type	AutoPose (10^3^/g)	Lipogems (10^3^/g)	p-value
Viable TNC	58 ± 35	12 ± 7	0.016
WBC: CD45+	7.0 ± 3.1	8.8 ± 6.6	0.276
Stromal MSC: CD45−31–90 +73 + 105+	51 ± 32	3 ± 2	0.015

Aggregate mean and standard deviation (SD) of data represented as “mean ± SD”.

### Cytokine and growth factor comparison

Of the 23-factors analyzed, 15 were below detection limits and excluded from analysis. These included PDGF-BB, IL-13, IL-33, IL-10, IL-4, TGFα, IL-15, IFN-α, IL-7, IL-3, GM-CSF, TNF-α, IL-1β, IL-1α, and IFN-γ. VEGF, TGF-β1, M-CSF, bFGF, IL-6 and IL-2 concentrations were significantly higher in AutoPose supernatants compared to Lipogems (p ≤ 0.01, Table 5) while bFGF was higher in Lipogems than Autopose (p = 0.03, Table 5).

**Table 5:** Growth factor and cytokine content in the cell culture supernatant after 7 days.

Cytokine/growth factor	AVG conc. (SD) (pg/g MFAT)		p-value
	Autopose	Lipogems	
**Anti-inflammatory**			
IL-1ra	46,341.8 ± 25,550.7	30,970.7 ± 14,968.7	0.106
VEGF*	4365.9 ± 1320.1	1916.9 ± 195.2	0.007
TGF-β1*	12,191.2 ± 1275.5	10,985.6 ± 1423.3	0.001
M-CSF*	95,261.4 ± 15,314.7	51,750.1 ± 19,268.4	0.000
HGF	32,719.8 ± 6936.0	33,205.6 ± 14,316.0	0.464
PDGF-BB	< 24.4	< 24.4	N/A
IL-13	< 58.5	< 58.5	N/A
IL-33	< 38.2	< 38.2	N/A
IL-10	< 88.3	< 88.3	N/A
IL-4	< 3.2	< 3.2	N/A
TGF-α	< 17.8	< 17.8	N/A
**Immunoregulatory**			
bFGF*	88.1 ± 50.7	271.1 ± 167.1	0.031
IL-6*	100,481.5 ± 35,989.2	54,585.9 ± 36,395.0	0.000
IL-2*	410.1 ± 29.2	316.3 ± 52.4	0.011
IL-15	< 7.7	< 7.7	N/A
IFN-α	< 12.3	< 12.3	N/A
IL-7	< 8.8	< 8.8	N/A
IL-3	< 34.2	< 34.2	N/A
**Proinflammatory**			
GM-CSF	< 25.3	< 25.3	N/A
TNF-α	< 25.1	< 25.1	N/A
IL-1β	< 9.5	< 9.5	N/A
IL-1α	< 27.1	< 27.1	N/A
IFN-γ	< 121.9	< 121.9	N/A

Aggregate mean and standard deviation (SD) of data represented as “mean ± SD”. When concentrations were below detectable limits, less than the limit of detection is shown and statistical analyses was not performed, indicated by “N/A”.

*Represents statistically significant difference between MFAT groups.

## Discussion

In this study, the AutoPose and Lipogems System were used to produce micro-fragmented adipose tissue (MFAT) using a combination of resizing and saline wash steps. It was found that relative proportions of cells and overall concentration of total nucleated cells varied based on MFAT system and analytical technique used. The heavier washing protocol used in the Lipogems system is attributed to the overall lower product yield compared to lipoaspirate input as well as lower TNC and stromal MSC concentrations. Generally, progenitors will remain within the ECM unless liberated by enzymatic or mechanical means^3^. It is possible that the final resizing step occurring in the presence of saline, liberated additional stromal cells which were then decanted, or that these cells are more sensitive to stresses from heavy washing. The Lipogems product tended to retain similar concentrations of EC and pericytes to the Autopose System and this may be due to tighter association or protection of these cells by the vessel walls. Concentration of WBCs in MFAT from each system was consistently similar, suggesting that the lighter washing protocol performs equally to the heavier washing protocol in reducing these cells from the product. RBC and oil concentrations were not evaluated which would likely be affected by washing regime.

Analytical techniques utilizing enzymatic digestion yielded higher numbers of TNC for analysis. This is consistent with other published findings that concluded that enzymatically digested adipose tissues yield approximately 5000 to 2,000,000 TNCs per gram^12–14^. Relative cell proportions in SVF of lipoaspirate have been shown to be anywhere from 5 to 30% adherent ASCs, 22–45% mature blood cells, and 10–25% cells of endothelial lineage^1,15,16^. In this study, comparable cell proportions were observed from the SVF isolated from MFAT when measured immediately, however the population shifted to be predominantly progenitor cell phenotype with a small proportion of endothelial cells after a short three-day culture period due the tissue culture adherent properties of these cells.

In contrast to SVF analysis, longer term culture of MFAT, not preceded by enzymatic digestion, resulted in a larger ratio of stromal MSCs compared to other cell types, demonstrating that plastic-adherent MSCs were able to migrate from the MFAT, while the majority of other cell types remained in the tissue or were washed away during culture. The overall magnitude of TNC number was lower by a factor of 10 as compared to enzymatic analysis. Initial evaluation of the cells liberated by enzymatic means eliminate potential effects of cell culture and therefore data may reflect more closely the representation of cells immediately present in the injectable product used at point-of-care treatment. Alternatively, cell outgrowth, specifically from the direct culture of MFAT, may indicate availability of stromal cells at the treatment site.

At early timepoints, the majority of CD45−CD34+ cells were also positive for CD90 and CD73. Positive CD105 expression was only observed after 3 days of culture similar to other studies^8^. The majority of stromal cells were observed to be CD146−, further differentiating the CD34+ MSC population from CD45−CD34−CD146+ pericytes, although CD146 was not included in the criteria for a stromal MSC (CD45−31−34+90+73+105+). Consistent with other published findings^6^, in the cultures evaluated at 7 days, only 0.5–40% (9.3 ± 13.7%) of the culture adherent CD45−, CD146−, CD90+, CD73+, CD105+ cells still expressed CD34. Therefore, CD34 was not used as a criteria to define MSC populations on Day 7. This contrasts with the majority of stromal MSC that were still positive for CD34 on Day 3.

Secretome analysis of unstimulated MFAT produced from both systems resulted in detection of anti-inflammatory cytokines/growth factors (IL-1ra, VEGF, TGF-β1, M-CSF, HGF) and immunoregulatory cytokines (bFGF, IL-6, IL-2). Both IL-1ra and VEGF are directly related to tissue healing and reduction of inflammation^17^. VEGF, TGF-β1, HGF, and bFGF are known angiogenic growth factors produced by adipose-derived progenitor and stromal cells^18,19^. IL-6 and IL-2 are immunomodulatory cytokines secreted by immune and stromal cells including T cells, monocytes/macrophages, fibroblasts, and endothelial cells^20,21^. Their complex role in immunoregulation could provide benefit in terms of macrophage polarization to an anti-inflammatory M2 state, which can facilitate healing, remove cell debris, and promote tissue repair^22,23^. Interestingly, the pro-inflammatory cytokines/growth factors (GM-CSF, TNF-α, IL-1β, IL-1α, IFN-γ) analyzed in this study were not detected. In contrast, isolated ASCs stimulated with lipopolysaccharide (LPS) have been shown to secrete high levels of IL-1β, IFN-γ, and TNFα^24^ relative to the levels of proinflammatory mediators observed in the present study. The presence of large quantities of IL-1ra known to inhibit the inflammatory cascade induced by IL-1 suggests the secretome produced by resized adipose tissue may be predominately anti-inflammatory^17^. This data presented represents MFAT in a simple culture system and additional studies are required to better understand how the more complex in vivo environment may affect the MFAT secretome.

One limitation is that the lipoaspirate was not collected using the harvest cannula provided with each kit. The samples were instead obtained as biproducts of lipoaspiration procedures that utilized harvesting cannulas with larger flutes. To control for the differences in tissue harvest, the lipoaspirate samples were passed through each system’s corresponding cannula to produce initial tissue sizes as contemplated within each system’s instructions for use. While our strategy provides a similar sample to be processed with each system, it is therefore possible that additional findings may have been observed if the initial sample collection was conducted using the specific cannulas provided with the Lipogems and AutoPose Systems. Normalizing results based on per weight of the initial adipose sample removed potential variation in pipetting a set volume of micro-fragmented adipose. Additionally, analysis of MFAT via flow cytometry poses the limitation of measuring autofluorescent contaminants within the cell populations, due to oil and cell debris contained in MFAT. To resolve this, a nuclei stain could be added to the composition panel for future studies.

## Conclusion

The results of this study demonstrate the MFAT produced by different systems is likely to differ in cellular content which can affect cytokine secretion. While the clinical implications of these differences are unknown, as with all autologous therapies, this study highlights the importance of product characterization. Furthermore, working toward standardization of product characterization techniques provides benefit for comparison of research studies, ultimately leading to optimization of adipose-derived treatments for therapeutic applications.

## Supplementary Information

Supplementary Figure 1.

Supplementary Figure 2.

Supplementary Figure 3.

Supplementary Figure 4.

Supplementary Figure 5.

## Data Availability

The datasets generated during and/or analyzed during the current study are available from the corresponding author on reasonable request.
