# Supplementary figures and images for: Micro-fragmented adipose tissue cellular composition varies by processing device and analytical method
Source: Sci Rep. 2022 Sep 27;12:16107. doi: 10.1038/s41598-022-20581-1 (PMC9515206; doi:10.1038/s41598-022-20581-1)

Time 0 Viable TNC Gating Example

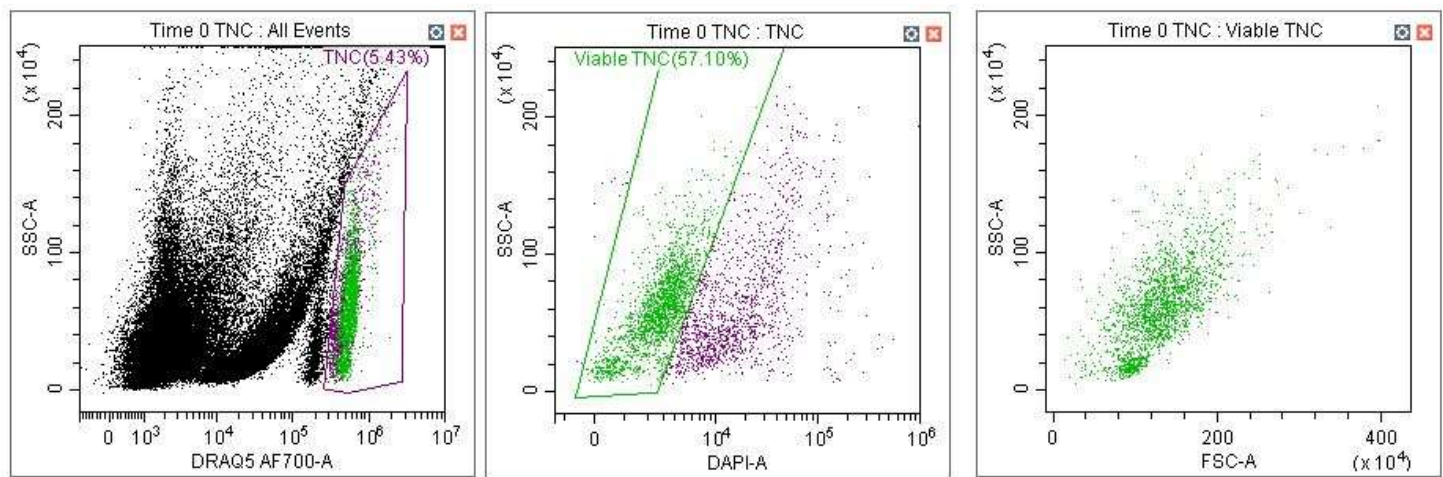

Day 3 Viable TNC Gating Example

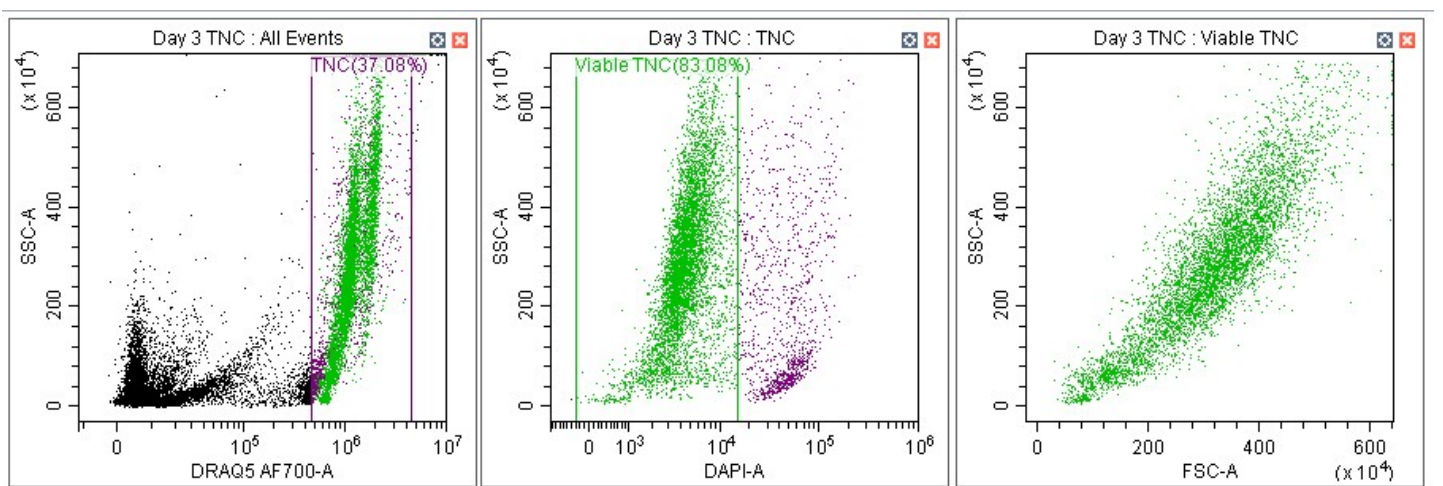

Day 7 Viable TNC Gating Example

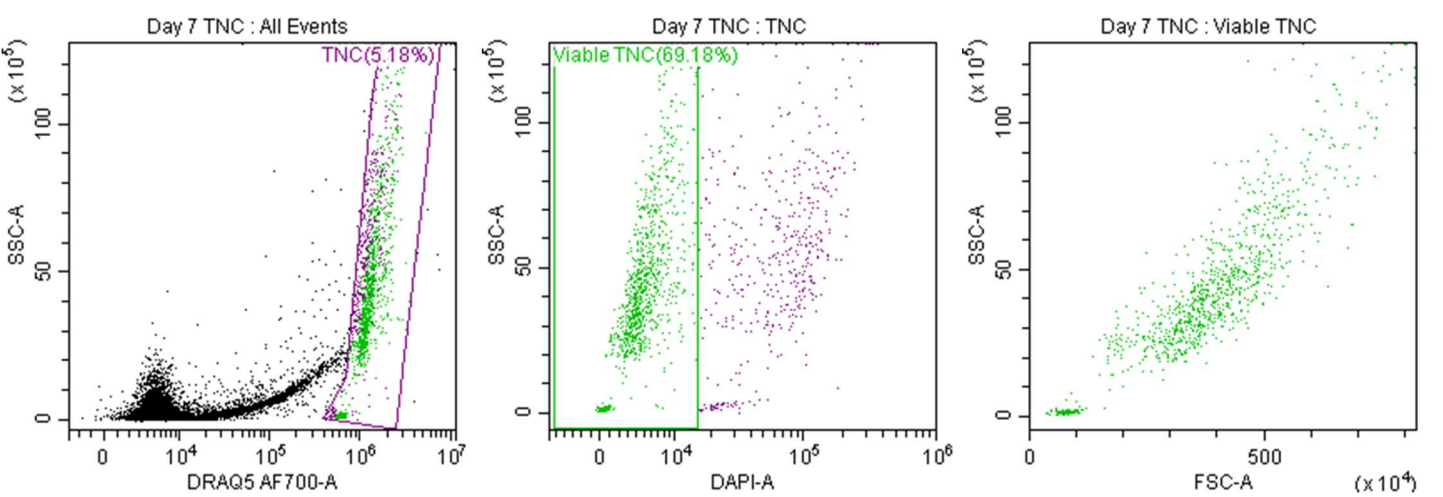

Supplement: Supplementary file 1 — Supplementary Figure 1. [file 41598_2022_20581_MOESM1_ESM.pdf]

Time 0 Cell Population Gating Example

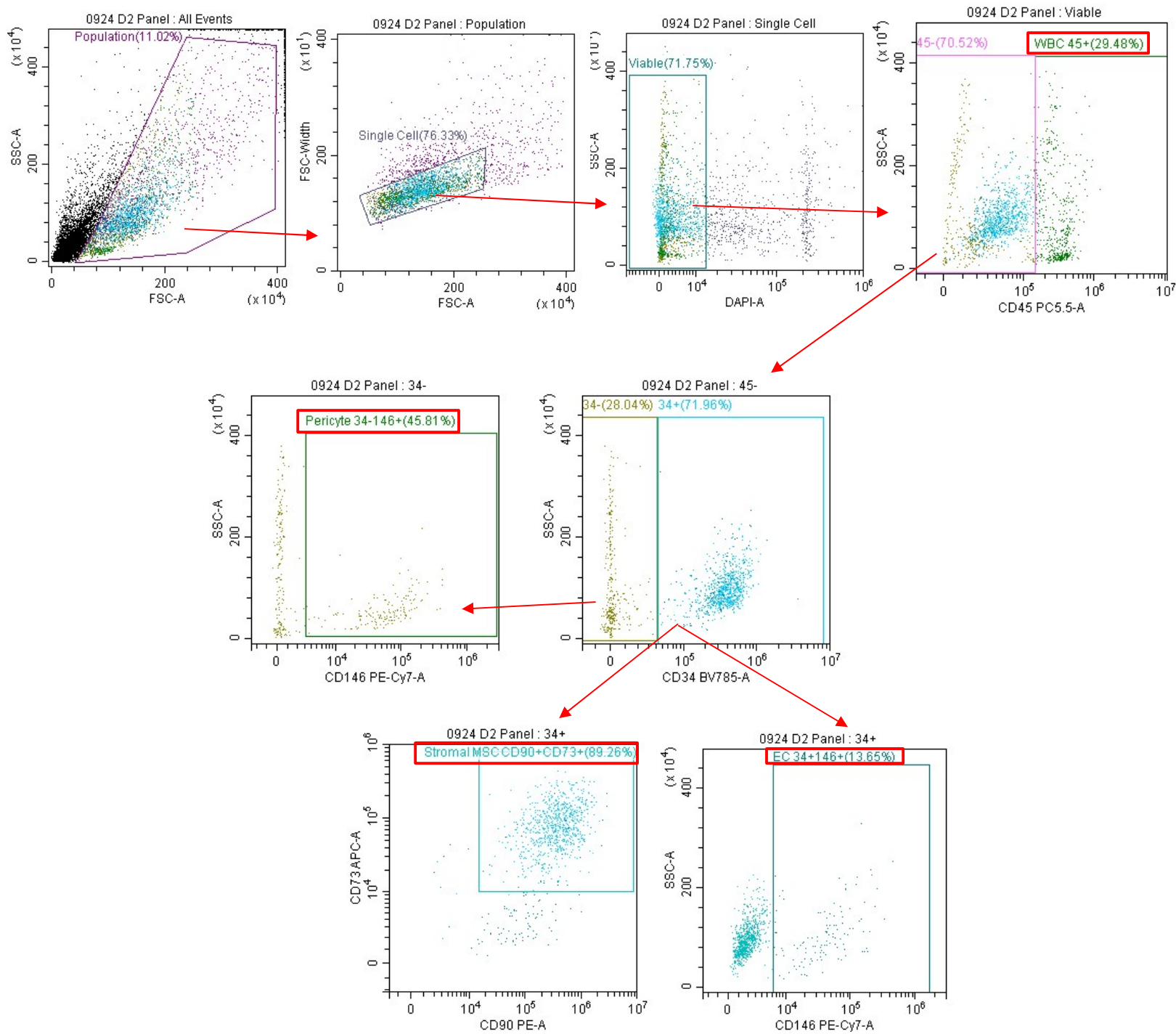

Supplement: Supplementary file 2 — Supplementary Figure 2. [file 41598_2022_20581_MOESM2_ESM.pdf]

Day 3 Cell Population Gating Example

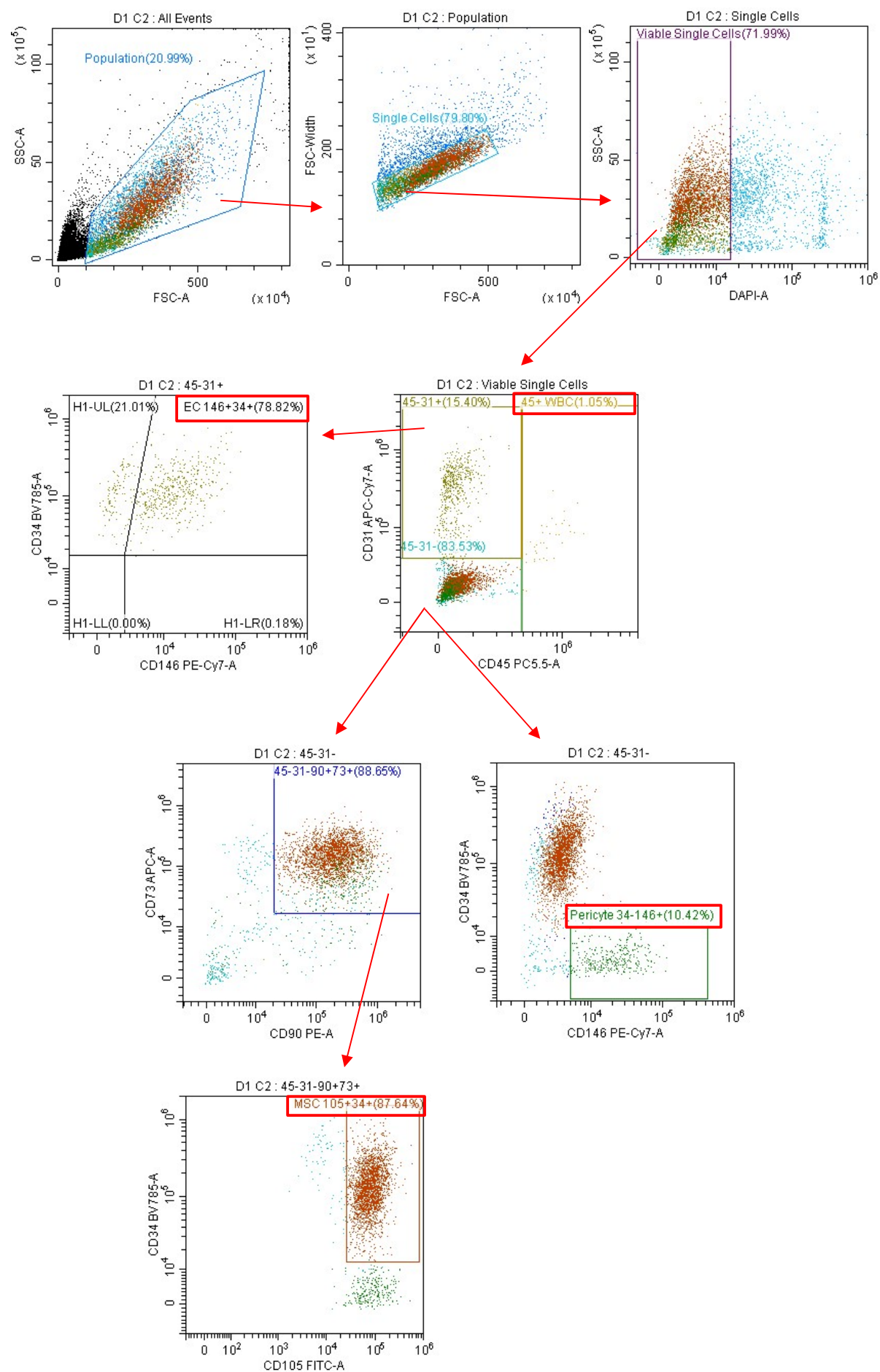

Supplement: Supplementary file 3 — Supplementary Figure 3. [file 41598_2022_20581_MOESM3_ESM.pdf]

## Day 7 Cell Population Gating Example

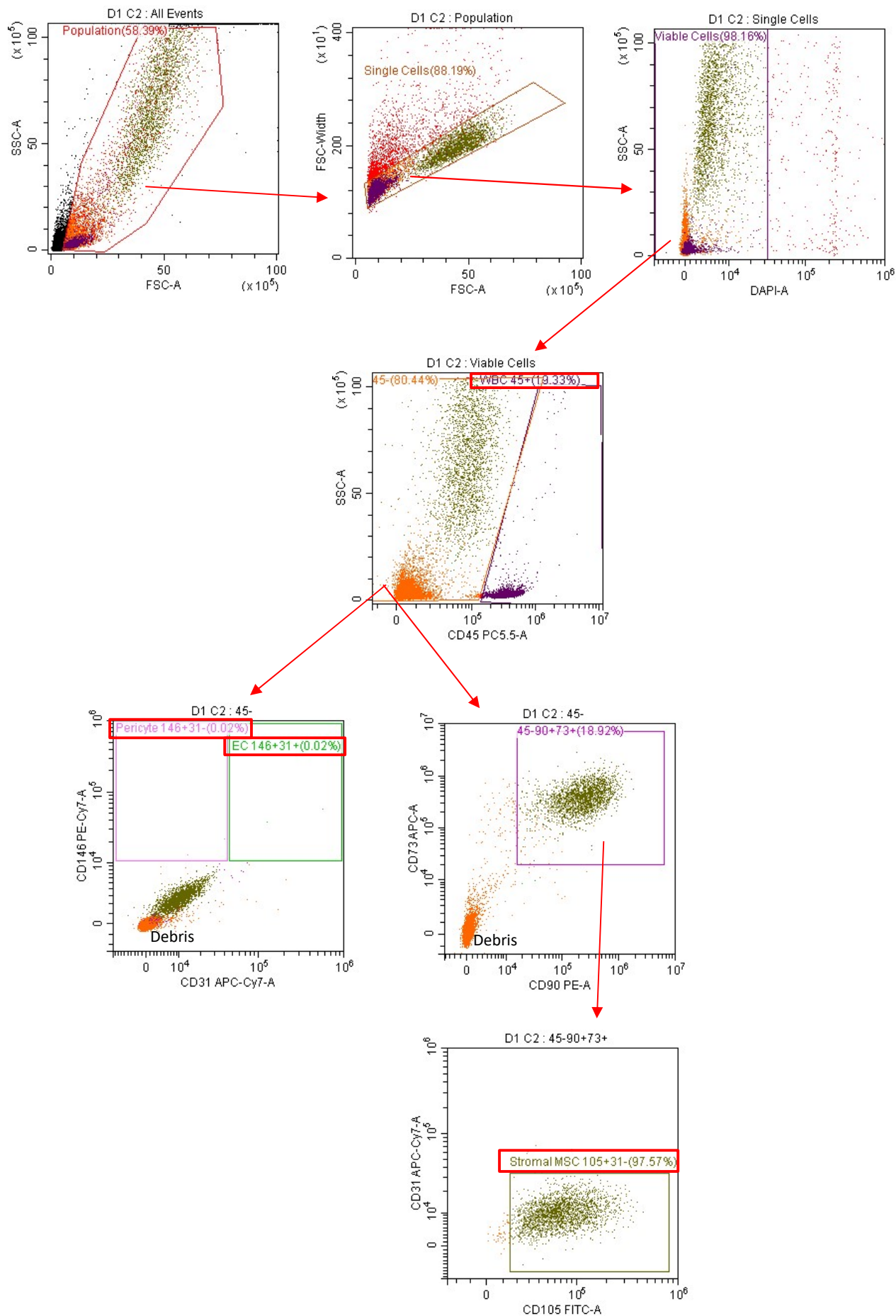

\*Orange population = debris

Supplement: Supplementary file 4 — Supplementary Figure 4. [file 41598_2022_20581_MOESM4_ESM.pdf]

## FMO Gating- All Markers

### CD31 & CD45 FMOs:

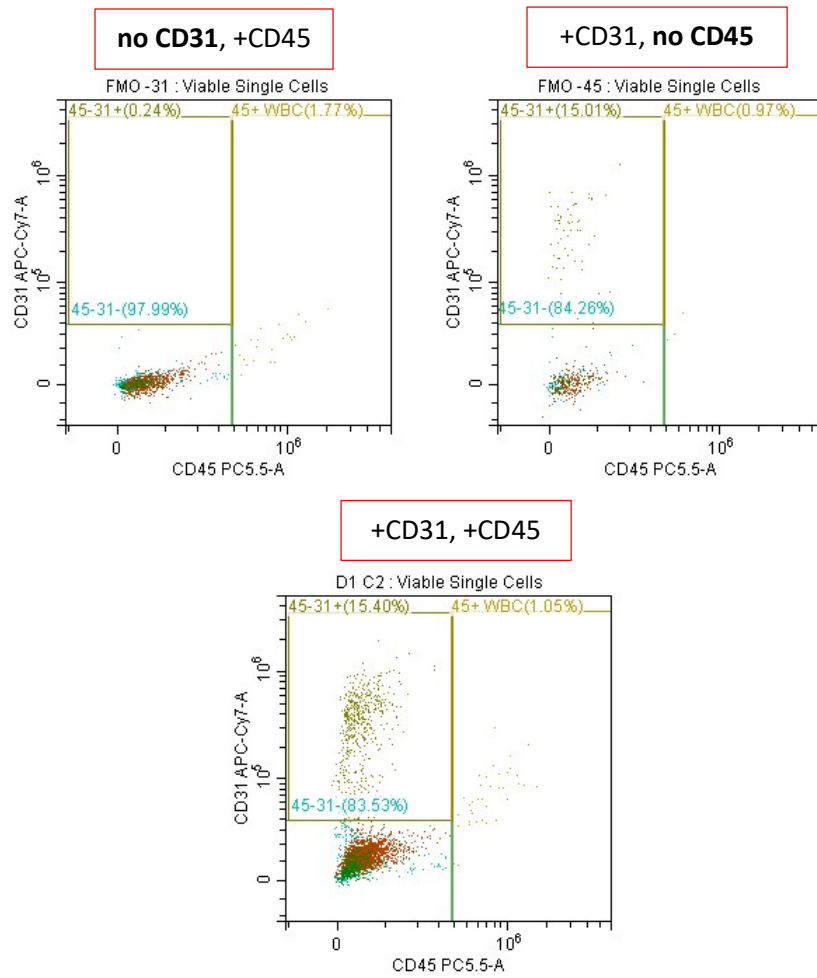

CD146, CD34, CD105 FMOs:

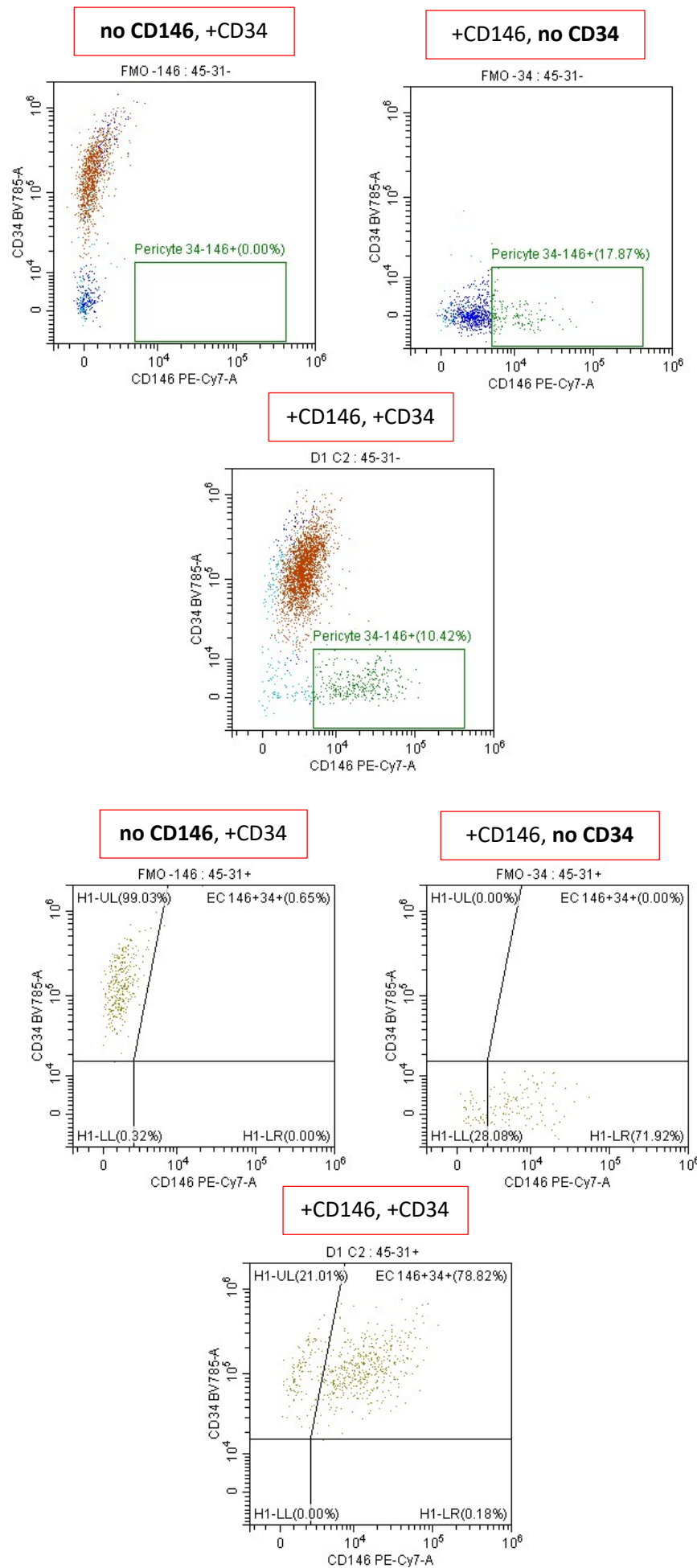

**no CD34, +CD105**

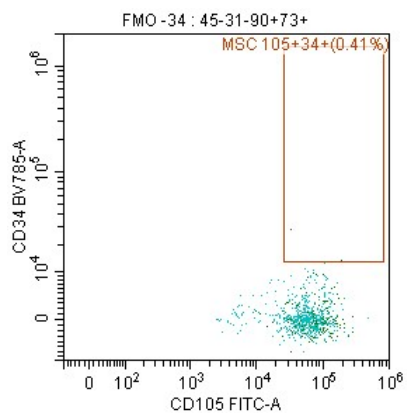

**+CD34, no CD105**

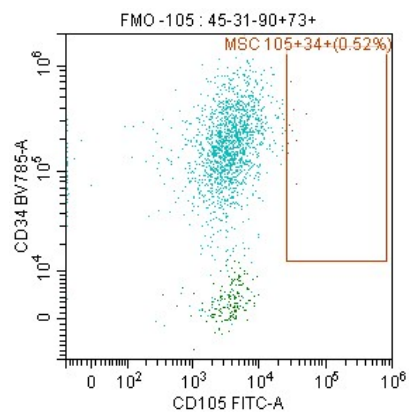

**+CD34, +CD105**

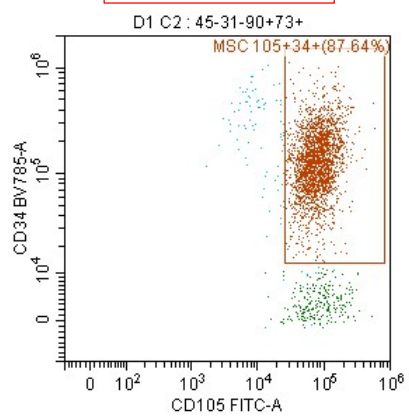

CD73 & CD90 FMOs:

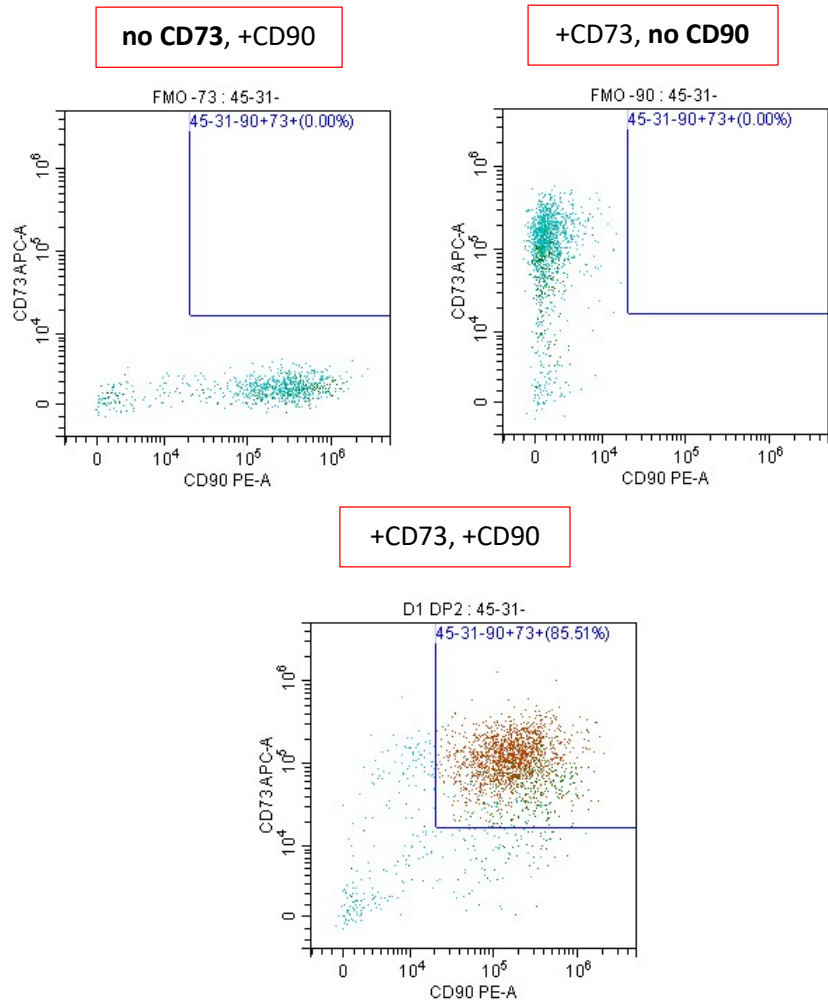

Supplement: Supplementary file 5 — Supplementary Figure 5. [file 41598_2022_20581_MOESM5_ESM.pdf]
